# Treatment with *Rhizoma Dioscoreae* Extract Has Protective Effect on Osteopenia in Ovariectomized Rats

**DOI:** 10.1155/2014/645975

**Published:** 2014-01-09

**Authors:** Zhiguo Zhang, Lihua Xiang, Dong Bai, Xiaowei Fu, Wenlai Wang, Yan Li, Hong Liu, Jinghua Pan, Ya'nan Li, Gary Guishan Xiao, Dahong Ju

**Affiliations:** ^1^Institute of Basic Theory, China Academy of Chinese Medical Sciences, Beijing 100700, China; ^2^Clinical Medical College of Chinese and Western Medicine, Shaanxi University of Chinese Medicine, Xi'an 712046, China; ^3^Functional Genomics and Proteomics Laboratory, Osteoporosis Research Center, Creighton University Medical Center, 601 North 30th Street, Suite 6730, Omaha, NE 68131, USA; ^4^Institute of Basic Theory, China Academy of Chinese Medical Sciences, No. 16 Nanxiaojie, Dongzhimennei, Beijing 100700, China

## Abstract

The aims of this study were to evaluate the osteoprotective effect of aqueous extract from *Rhizoma Dioscoreae* (RDE) on rats with ovariectomy- (OVX-) induced osteopenia. Our results show that RDE could inhibit bone loss of OVX rats after a 12-week treatment. The microarray analysis showed that 68 genes were upregulated and that 100 genes were downregulated in femurs of the RDE group rats compared to those in the OVX group. The Ingenuity Pathway Analysis (IPA) showed that several downregulated genes had the potential to code for proteins that were involved in the Wnt/**β**-catenin signaling pathway (Sost, Lrp6, Tcf7l2, and Alpl) and the RANKL/RANK signaling pathway (Map2k6 and Nfatc4). These results revealed that the mechanism for an antiosteopenic effect of RDE might lie in the synchronous inhibitory effects on both the bone formation and the bone resorption, which is associated with modulating the Wnt/**β**-catenin signaling and the RANKL/RANK signaling.

## 1. Introduction

Osteoporosis, the most common bone remodeling disease, is defined as a low bone mass and a high risk of fractures. Osteoporosis mainly affects postmenopausal women and elderly men. Osteoporosis is caused by an abnormal bone remodeling, that is, an excess of resorption and less formation, thereby resulting in an increased risk of hip and vertebral fractures [1, 2]. The development of bone fragility in postmenopausal women resulted from rate changes in bone remodeling, leading to alterations of the trabecular bone volume and architecture [3]. In rats, ovariectomy- (OVX-) induced bone loss can be treated with estradiol. Because rats and humans share similarities in skeletal responses to estrogen deficiency, the mature OVX rat is considered to be a suitable animal model for studying early postmenopause-induced bone loss [4].

Estrogen, bisphosphonates, parathyroid hormone (PTH), or selective estrogen receptor modulators (SERMs) have been used to prevent the postmenopausal bone loss [5], but many lines evidence indicate that long-term treatments with those drugs might cause adverse reactions, such as an increased risk of ovarian and endometrial cancer [6–9], osteonecrosis of the jaws [10], nervous system disorders [11], and venous thromboembolism [12]. Thus, an alternative therapeutic strategy with a proven efficacy and safety should be developed to prevent and treat osteoporosis. For years, Chinese herbal medicine has been widely used in clinical practice to treat bone diseases and will most likely continue to be used as a cost-effective alternative medicine in China [13]. *Rhizoma Dioscoreae* (RD) has been used to strengthen bone for a long time in China. However, the potential therapeutic effect of RD on osteopenia induced by estrogen deficiency has not been established. The objectives of this study were to evaluate the effect of the aqueous extract from RD (RDE) on ovariectomized rats and to explore the molecular targets of RDE.

## 2. Materials and Methods

### 2.1. Preparation of Aqueous Extract

RD is the dried rhizoma of *Dioscorea opposita* Thunb., which is produced in China. RD was purchased from Taizhou Medicinal Herbs Co. Ltd. (Zhejiang, China) and identified and authenticated by an expert herbalist at the Institute of Chinese *Materia Medica*, China Academy of Chinese Medical Sciences (CACMS).

The raw RD (250 g) was boiled twice with 4 L of distilled water for two hours under reflux. The aqueous extract was collected and filtered. The filtrates were concentrated under reduced pressure at 50°C and lyophilized into powder. The extractions from the two herbs yielded approximately 20% (w/w).

### 2.2. High-Performance Liquid Chromatography (HPLC) Analysis

The antiosteopenic compounds in RDE have not been identified completely until now. We used markers to identify and authenticate herbs and to analyze the chemical characteristics of RDE using HPLC. We ordered the standard samples of markers, diosgenin and allantoin, from the National Institutes for Food and Drug Control (Beijing, China).

Diosgenin and allantoin are used as markers to identify and authenticate RD according to the relevant literature [14–16].

For HPLC detection of the diosgenin, we used Agilent SB-C18 HPLC column (4.6 × 250 mm, 5 *μ*m, Zorbax, Agilent, Santa Clara, CA, USA) and DAD detector. The separating conditions were as follows: mobile phase, methanol : water (90 : 10, V/V), and flow rate, 1.0 mL/min. The chromatograms were recorded at 210 nm by monitoring spectra within a wavelength range of 190 to 600 nm. The separating conditions of allantoin were as follows: mobile phase, methanol : water (1 : 99, V/V), and flow rate, 0.5 mL/min. The chromatograms were recorded at 224 nm.

**Figure 1:**
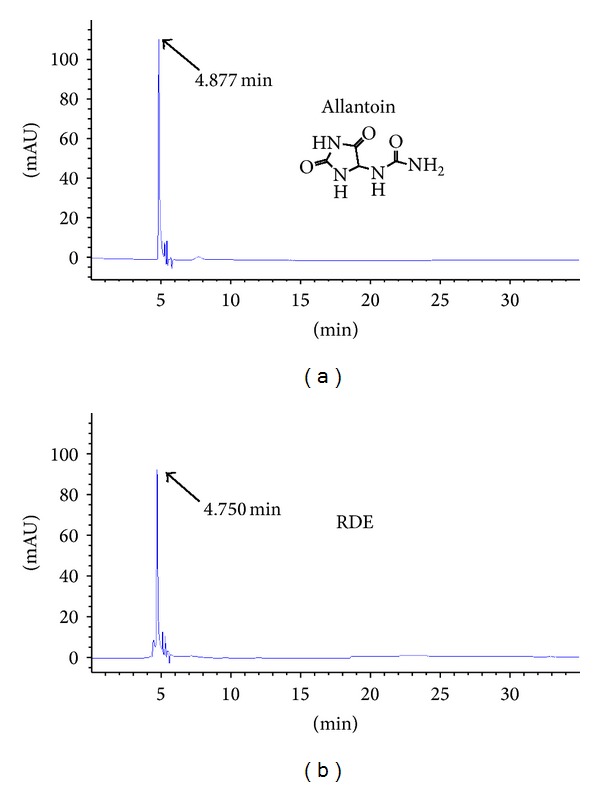
HPLC for the qualitative analysis of allantoin in the RDE. The chromatograms for standard substances and RDE are shown in (a) and (b), respectively. ((a) and (b)) The chromatograms for standard substances of allantoin and RDE, respectively.

**Figure 2:**
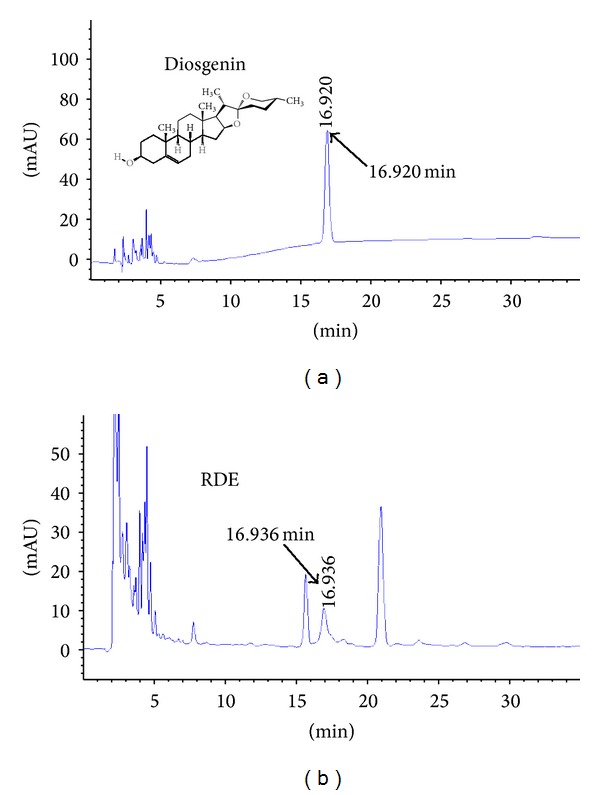
HPLC for the qualitative analysis of diosgenin in the RDE. The chromatograms for standard substances and RDE are shown in (a) and (b), respectively. ((a) and (b)) The chromatograms for standard substances of diosgenin and RDE, respectively.

Figures 1 and 2 present the typical chromatographic profile, which includes standard samples and RDE. Diosgenin and allantoin were adequately resolved from other unknown compounds and could be clearly identified by retention time. The content of the two compounds in the extract was calculated from the relevant peak areas by an external standard method. The percent of diosgenin and allantoin in the RDE was quantified as 0.52% and 1.46%, respectively.

### 2.3. Animal Grouping and Treatments

Many studies had shown that 6-month-old female rat ovariectomized bilaterally was a good model for postmenopausal osteoporosis [17–19]. We obtained a total of 48 6-month-old virgin Wistar rats with body weight of 310 ± 20.0 gram from the Experimental Animal Center of Academy of Military Medical Sciences (SCXK-(Military) 2002-001, Beijing, China). The Institutional Ethics Committee of CACMS approved the experimental research on the animals. The acclimatized rats were either Sham-operated (SHAM, *n* = 12) or bilaterally ovariectomized (OVX, *n* = 36) using the dorsal approach [20]. The OVX rats were randomly divided into three groups: OVX group (OVX, *n* = 12); 17*β*-estradiol treatment group (E2, *n* = 12); RDE group (RDE, *n* = 12). The rats in the E2 group received 17*β*-estradiol (Sigma-Aldrich, Saint Louis, MO, USA), which was dissolved in small amounts of ethanol with the volume adjusted with olive oil, to produce a concentration of 30 *μ*g/kg body weight, which was administered daily subcutaneously. The rats in the RDE group was fed standard rodent chow (Animal Center of the Fourth Military Medical University, Xi'an, China) and RDE at 1.3 g/kg body weight/day (dissolved in distilled water) by oral gavage. The gavage dosage was used based on the recommended dosage for humans (30 g/day) according to Chinese Pharmacopeia, multiplied by the rat/human body mass ratio. We used 13-fold (1.3 g/kg body weight/day) of the adult dosage as dosage of rats. The rats in the SHAM and the OVX groups were administered the same volume of distilled water by oral gavage. The treatment started 1 week after surgery for 12 weeks. On the 15th day and the 3rd day before sacrifice, all of the rats received tetracycline (Sigma-Aldrich, Saint Louis, MO, USA) at 30 mg/kg body weight by intraperitoneal injection.

### 2.4. Preparation of Specimens

One day after the last treatment, the animals were anesthetized with ketamine at 80 mg/kg body weight together with xylazine (12 mg/kg body weight, intraperitoneally injected) and sacrificed by exsanguination. We obtained blood samples by puncturing the abdominal aorta before death; we collected the blood samples in heparinized tubes. The blood samples were centrifuged at 3,000 ×g at 4°C for 10 min, aliquoted, and frozen at −80°C until the samples were used for assay. The proximal right tibiae were dissected and filled with physiological saline and stored at −20°C for measurements of bone mineral density (BMD) and microstructure by microcomputerized tomography (micro-CT). After these measurements were determined, the proximal right tibiae were fixed in 4% paraformaldehyde for 24 h, dehydrated in an ethanol gradient of 80%, 90%, and 100% for 2 days at each step, defatted in xylene for 2 days, and embedded in plastic polymer according to a method previously described [21]. Each undecalcified sample was sliced into two 5 *μ*m longitudinal sections with microtome (Reichert-Jung 2040, Germany). Undecalcified sections were stained with methylene blue or used for fluorescence morphology observation. The distal right femurs were dissected and stored at −80°C for microarray and real-time PCR assays.

### 2.5. BMD Assessment

The BMD of the tibiae were measured using OSTEOCORE 3 Visio by DXA (Medilink, Montpellier, France) according to a method previously described [22].

### 2.6. Bone Histomorphometric Analysis

Undecalcified tibial sections were used for measuring bone metabolism. All of the measurements were performed using the automated upright microscope system (Leica DMB6000B and CTR6000, Leica, Wetzlar, Germany) and image analysis system (Qwin, Leica, Wetzlar, Germany). The five bone histomorphometric indices on bone mass and bone turnover were analyzed, including the trabecular bone volume (BV/TV), eroded surface (ES/BS), active forming surface (MS/BS), mineral apposition rate (MAR), and osteoid seam width (O.Th). All of the histomorphometric indices were reported according to the standardized nomenclature recommended by the American Society of Bone and Mineral Research [23]. The animal data were obtained by blind measurements.

### 2.7. Biochemical Markers of Bone Turnover

The serum levels of bone formation marker, procollagen type 1 N-terminal propeptide (PINP), and bone resorption marker, C-terminal cross-linked telopeptides of type I collagen (CTX), were assessed using enzyme-linked immunosorbent assay (Immunodiagnostic Systems Ltd., Boldon, UK) for control, standard, and duplicate tests. Absorbance was read using an ELISA reader (Bio-Tek, Colmar, France) at 450 nm.

### 2.8. Micro-CT Analysis

The right proximal tibia of each animal, without sample preparation or decalcification, was scanned with a high-resolution micro-CT (Skyscan 1172 micro-CT system, Antwerp, Belgium). The Skyscan micro-CT uses the cone-beam reconstruction method to determine the conical geometry of the X-ray source. The desktop Skyscan micro-CT system was used according to a method previously described [24]. Each sample was placed on the rotational stage and translated along the continuously variable magnification stage to achieve the desired resolution (6.8 *μ*m). The sample was rotated through 185° and the images were made every 0.9°. Repeated scans were performed in the beginning of the experiments to verify the reproducibility of the method. A low-pass filter was used to remove the noise of the resulting gray-scale images. The trabecular bone was determined by a fixed threshold.

After the images were captured (100 keV, 100 *μ*A), a cube (1.5 mm × 1.5 mm × 1.5 mm) beginning 1 mm beneath the growth plate of the tibia was established as the “volume of interest” (VOI). We performed morphological measurements of the trabecular bone for the VOI using the standard Skyscan software package. We used three-dimensional analyses to assess the bone volume fraction (BV/TV), the trabecular thickness (Tb.Th), the trabecular separation (Tb.Sp), the trabecular number (Tb.N), the structural model index (SMI), and the degree of anisotropy (DA) for the same VOI [25].

### 2.9. Microarray Data Analysis

We performed a microarray analysis of whole-genome gene expression profiling using NimbleGen chips (Roche) that consisted of 26,419 probes for rat genes by KangChen Bio-tech. The total RNA of distal right femurs from RDE group and OVX group was harvested using TRIzol (Invitrogen) and the RNeasy kit (Qiagen) according to the manufacturer's instructions, including a DNase digestion step. After RNA measurement using the NanoDrop ND-1000 and denaturing gel electrophoresis, we amplified and labeled the samples using a NimbleGen One-Color DNA Labeling Kit. After hybridization in the NimbleGen Hybridization System and washing, the processed slides were scanned with the Axon GenePix 4000B microarray scanner. The data files were imported into Agilent GeneSpring Software (Agilent, version 11.0) for analysis. The gene expression was normalized to the OVX group. The differentially expressed genes were identified through the fold change and *t*-test *P* value screening.

### 2.10. Ingenuity Pathway Analysis

To facilitate the gene microarray data analysis and relate specific genes to the underlying biological processes, IPA (Ingenuity Systems, Redwood, CA, USA) (http://www.ingenuity.com/) was used for the function and pathway analysis. Differentially expressed genes between the RDE group and the OVX group were imported into IPA and the top five canonical pathways were observed. The canonical pathway analysis identified the molecular pathways from the IPA library of canonical pathways that were most significant to the dataset. The genes from the dataset that were associated with a canonical pathway were considered for additional analysis.

### 2.11. Quantitative Real-Time- (RT-)PCR (qPCR)

The total RNA was purified using an RNeasy Mini Kit (Qiagen, Valencia, CA, USA), and 4 *μ*g RNA was reverse-transcribed using the Superscript First Strand synthesis system (Invitrogen, Carlsbad, CA, USA) to cDNA. The qPCR amplification was performed using the SYBR-green detection of PCR products in real time with an ABI-7500 Sequence Detection System (Applied Biosystems, Foster City, CA, USA) according to the manufacturer's instruction. The primers used in the qPCR analysis are presented in Table 1. The PCR program was initiated by 10 s at 95°C before 40 thermal cycles, each of 5 s at 95°C and 34 s at 60°C. We analyzed the data according to the 2^−ΔΔCt^ method and normalized to the glyceraldehyde-3-phosphate dehydrogenase (GAPDH) expression in each sample. The melting curves for each PCR reaction were generated to ensure the purity of the amplification product. A no-template negative control was included in each experiment.

### 2.12. Statistical Analysis

All values were expressed as the mean ± standard deviation. All analyses were conducted using the SPSS 13.0 (SPSS Inc., Chicago, IL, USA). The difference between the groups regarding the evaluated parameters was tested using the analysis of variance (ANOVA) followed by the Tukey test. The data of all groups passed the Kolmogorov-Smirnov test of normality. *P* < 0.05 was considered to be statistically significant.

## 3. Results

### 3.1. Effect of RDE on BMD and Bone Histomorphometric Indices

As shown in Table 2, ovariectomy decreased BMD substantially compared to the Sham-operated animals, and treatment with RDE for 12 weeks significantly increased BMD. E2 increased BMD almost completely to the level of the SHAM group.

The BV/TV was significantly reduced in rats with estrogen deficiency induced by ovariectomy. However, MS/BS, ES/BS, MAR, and O.Th were increased significantly in the OVX rats (Table 2). The E2 treatment significantly relieved the effects of ovariectomy on the histomorphometric indices by increasing BV/TV and decreasing MS/BS, ES/BS, MAR, and O.Th in the OVX rats. Compared to the OVX rats, the RDE treatment showed a similar effect to the E2 treatment on four indices except O.Th (Table 2).

### 3.2. Effect of RDE on Biomarkers of Bone Turnover

The serum levels of bone formation (PINP) and bone resorption (CTX) biomarkers after a 12-week treatment of different rat groups are provided in Table 3. At the end of week 12, the OVX group showed a significantly higher concentration of PINP or CTX than the SHAM group (*P* < 0.01). Moreover, the E2 and RDE group showed a significantly lower (*P* < 0.01 or *P* < 0.05) level of PINP or CTX than the OVX group (Table 3).

### 3.3. Effect of RDE on Trabecular Bone Microarchitecture

**Figure 3:**
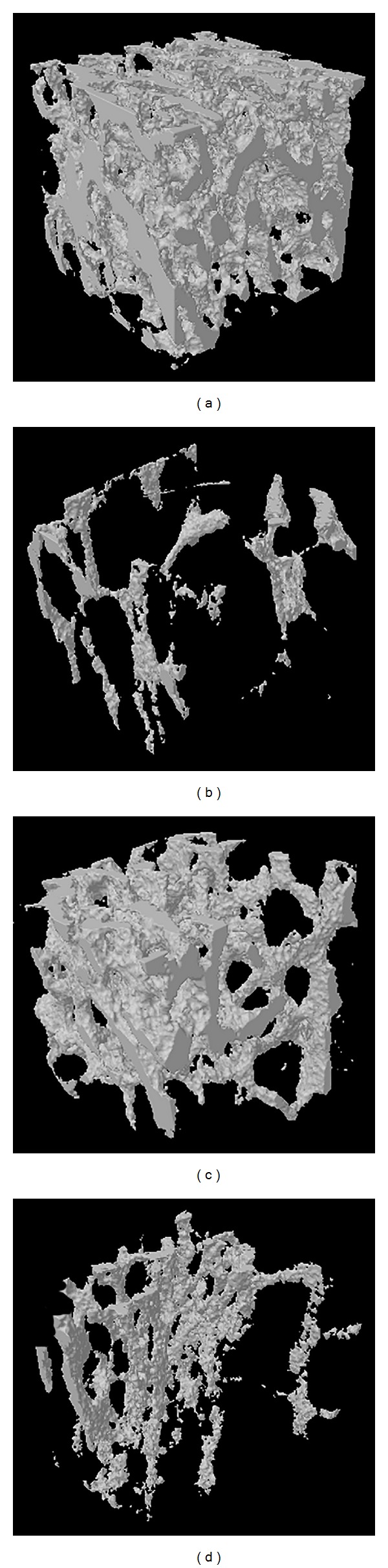
Representative sample from each group (*n* = 12 in each group): the 3D architecture of the trabecula bone beneath the tibial growth plate. (a) SHAM; (b) OVX; (c) E2; (d) RDE.

We further evaluated the trabecular bone microarchitecture of four groups. An analysis of the tibia morphometric parameters indicated that ovariectomy significantly decreased the trabecular BV/TV, Tb.N, and Tb.Th (*P* < 0.01) and increased Tb.Sp, SMI, and DA (*P* < 0.01) compared to the SHAM group. Compared to the OVX group, the treatment with RDE or E2 prevented the above mentioned findings significantly to some degree except SMI (Table 4). Figures 3(a)–3(d) show that the E2 or RDE treatment could relieve the damage of the trabecula induced by the ovariectomy.

### 3.4. Effect of RDE on Gene Expression Profile

The array results demonstrated that a total of 168 genes had altered the expression levels (≥1.5-fold) between the distal right femurs from RDE and OVX group rats; that is, 68 genes were upregulated and 100 genes were downregulated (see Supplementary Materials available online at http://dx.doi.org/10.1155/2014/645975). The differential expression genes are listed in Supplementary Materials.

### 3.5. Pathway Analysis of Differentially Expressed Genes

**Figure 4:**
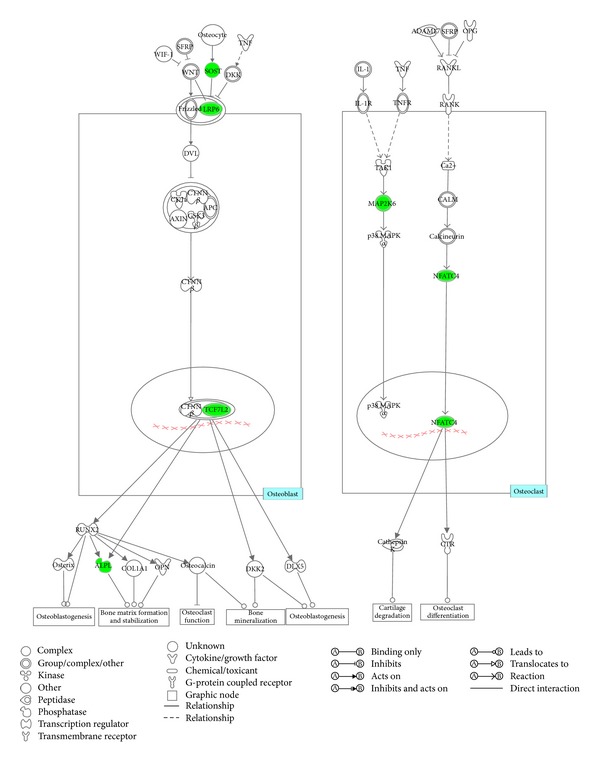
Schematic diagram illustrating the role of osteoblasts and osteoclasts in an antiosteopenic effect of RDE. The downregulated genes appear in green. The white colour indicates the genes that are not user specified but are incorporated into the network through relationships.

**Figure 5:**
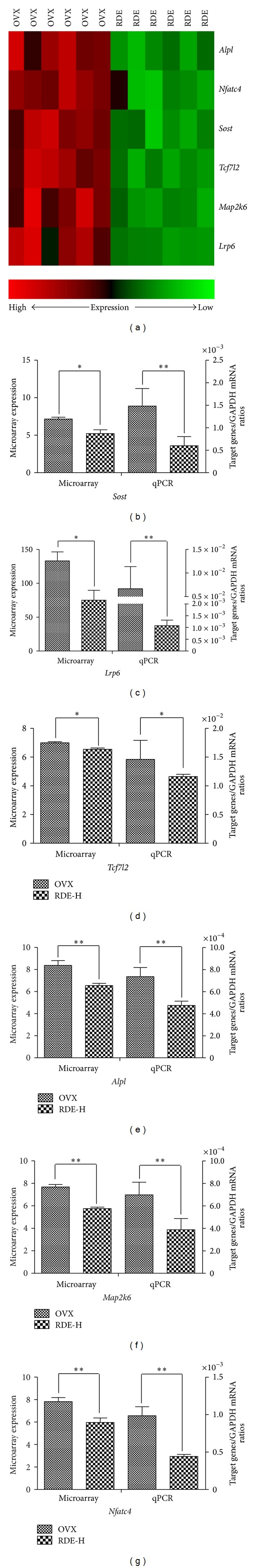
Validation of 6 differential expression genes identified by microarray and IPA in a replicated experiment by real-time qPCR. (a) Comparative changes in 6 genes as determined by microarray and qPCR (*n* = 6 in each group); (b)–(g) effect of RDE on the expressions of *Sost, Lrp6, Tcf7l2, Alpl, Map2k6*, and *Nfatc4*, respectively. *P < 0.05; **P < 0.01, compared with the OVX group.

We characterized differential expression genes into functions and signaling pathways by using IPA software according to Ingenuity Pathways Knowledge Base (IPKB) (http://www.ingenuity.com/). These signaling pathways were ranked according to the IPA calculated scores, which are based on the significance of the involved genes. Based on IPKB, the signature genes were classified into pathways by IPA. Among these, only one pathway was identified by IPA with significance values of less than or equal to 0.05 (Table 5). In the present study, we selected the following genes, sclerostin (*Sost*), low density lipoprotein receptor-related protein 6 (*Lrp6*), transcription factor 7-like 2 (*Tcf7l2*), alkaline phosphatase (*Alpl*), Mitogen-activated protein kinase kinase 6 (*Map2k6*) and nuclear factor of activated T cells, cytoplasmic, calcineurin dependent 4 (*Nfatc4*) involved in the significant signaling pathway “role of osteoblasts, osteoclasts, and chondrocytes in rheumatoid arthritis” (Figure 4) for additional evaluation. We found that all of the selected genes were downregulated in the RDE treated group rats (Figure 5).

### 3.6. Confirmation of Differential Levels of Gene Expression by qPCR

To confirm the differential gene expression detected by the microarray analysis and pathway analysis, we sampled six genes (*Sost, Lrp6, Tcf7l2, Alpl, Map2k6*, and *Nfatc4*) involved in the significant signaling pathway “role of osteoblasts, osteoclasts, and chondrocytes in rheumatoid arthritis” for verification using real-time PCR. Figures 5(b)–5(g) present comparative changes in these genes as determined by microarray and RT-PCR. In all of these cases, the results from qPCR generally agreed with the changes in the microarray analysis, although the absolute degree of change differed markedly between both methods.

## 4. Discussion

RD and other traditional Chinese tonic herbs have been used for many years to treat bone diseases in China. Several studies indicated that *Dioscorea alata* L. cv. and *Monascus*-fermented dioscorea had antiosteopenic effects [14, 26]. However, whether RDE has an antiosteopenic effect and whether the mechanism of the antiosteopenic effect is functional *in vivo* have not been established. In the present study, for the first time, we evaluated the effect of RDE on rats with osteopenia induced by OVX.

With an ovariectomy, BMD markedly decreases due to an increase in bone turnover in the OVX rats compared to the Sham rats, whereas the treatment with the RDE increased the BMD of the tibia compared to the OVX group.

We had used the indices of bone histomorphometry and the bone turnover biomarkers to explain the change in BMD. According to our experimental results, the OVX led to significant tibial bone loss, as shown by BV/TV, an important bone-mass index. Furthermore, coincident and significant increases in the indices for the assessment of bone resorption, ES/BS, and CTX and for the assessment of bone formation, MS/BS, MAR, O.Th, and PINP indicated that the mature OVX rat is a reliable and suitable animal model for studying the high turnover bone loss, such as early postmenopausal osteoporosis. The treatment with E2 or RDE for 12 weeks was able to prevent the bone loss induced by OVX, which was reflected by the increase in BV/TV, and lowered the increased bone turnover, which was reflected by the significant decreases in ES/BS, MS/BS, MAR, CTX, and PINP. RDE exhibited an inhibitory effect on the increased bone turnover. Furthermore, we assessed the trabecular 3D bone microarchitecture by micro-CT to confirm this effect. The results showed that the rat tibia in the RDE group underwent less bone loss than the OVX group, which was reflected by the significant change in BV/TV, Tb.Th, Tb.N, Tb.Sp, and DA.

To explore the mechanism of an antiosteopenic effect of RDE, we screened the differential expression genes with microarrays and found a key pathway with IPA. The microarray and IPA technology might provide a tool for new biomaterial development and clinical treatment.

In our study, we succeeded in identifying 168 genes, which were differentially expressed in the femur of the RDE group compared to the OVX group. Table 5 shows the top five canonical pathways from the hundreds of canonical pathways. We clarified the most important signaling pathway related to osteoblasts and osteoclasts (Figure 4), which is the most meaningful in transcriptome analysis. We observed that the Wnt/*β*-catenin signaling pathways were downregulated in osteoblasts after the RDE treatment (Figure 4).

The canonical Wnt/*β*-catenin signaling pathway led to osteoblast proliferation and bone matrix formation, stabilization, and mineralization [27, 28]. Sclerostin (SOST) was a potent inhibitor of the Wnt/*β*-catenin signaling pathway. Sclerostin is a glycoprotein, which is secreted by osteocytes. Sclerostin after secretion by osteocytes travels through the osteocyte canaliculi to the bone surface where it binds to the coreceptors low density lipoprotein receptor-related protein 5 (LRP5) and LRP6, thus preventing colocalization with frizzled protein and Wnt signaling, thereby reducing osteoblastogenesis and bone formation [29]. In the canonical Wnt/*β*-catenin signaling pathway, the T cell factor/lymphoid enhancer-binding factor (TCF/LEF) family transcription factors (e.g., TCF7L2) played a pivotal role in promoting bone-specific gene expression [30], such as alkaline phosphatase (ALP), which participated in the regulation of osteoblastic cell differentiation, proliferation, and migration [31].

We found that gene expressions of *Sost*, *Lrp6*, *Tcf7l2*, and *Alpl* in the femur were all downregulated after treatment of the RDE (Figure 5(a)). The downregulation of *Sost* could promote osteoblastogenesis, but the inhibitory effect on osteoblastogenesis caused by the downregulation of *Lrp6*, *Tcf7l2*, and *Alpl* might be predominant. Our qPCR analyses of *Sost*, *Lrp6*, *Tcf7l2*, and *Alpl* expressions (Figures 5(b)–5(e)) convinced us that RDE could decrease the high bone formation in the femur of OVX rat, which results from ovariectomy by attenuating the canonical Wnt/*β*-catenin signaling and that the results of the microarray and IPA were credible.

In Figure 4, we clarified that the most meaningful signaling pathway related to osteoclasts was the receptor activator of the NF-*κ*B ligand (RANKL)/receptor activator of the NF-*κ*B (RANK) signaling pathway. Osteoclasts were cells of monocyte and macrophage origin that degraded the bone matrix. RANKL, a tumor necrosis factor (TNF) family cytokine, induced the differentiation of osteoclasts in the presence of macrophage colony-stimulating factor. RANKL activated the TRAF6, c-Fos, and calcium signaling pathways, all of which were indispensable for the induction and the activation of the nuclear factor of activated T cells c1 (NFATC1), the master transcription factor for osteoclastogenesis. The autoamplification of the NFATC1 resulted in the efficient induction of osteoclast-specific genes [32, 33]. NFATc4 was an indispensable mediator of NFATc1 when NFATc1 exerted its osteoclastogenic effect [34].

A body of evidence suggested that the p38 mitogen-activated protein kinase (p38 MAPK) signaling pathway was a downstream of the RANK signaling pathway and played an important role in the osteoclast differentiation [35, 36]. The expression of dominant negative forms of p38 MAPK in RAW264.7 cells inhibited RANKL-induced differentiation of these cells into osteoclasts [37]. MAP2K6 is a direct and specific activator of all p38 MAPK isoforms identified to date [38].

We found that expressions of *Map2k6* and *Nfatc4* in the femur were all downregulated after treatment with RDE (Figure 5(a)). Our qPCR analyses of *Map2k6* and *Nfatc4* expressions (Figures 5(f)-5(g)) convinced us that RDE could decrease high bone resorption in the femur of OVX rat, which results from ovariectomy by attenuating RANKL/RANK signaling and that the results of the microarray and IPA were credible.

We observed that expressions of *Sost*, *Lrp6*, *Tcf7l2*, *Alpl*, *Map2k6*, and *Nfatc4* in the femur were all downregulated after the treatment with RDE (Figure 5). On the one hand, the downregulation of *Sost* could promote osteoblastogenesis, but the inhibitory effect on osteoblastogenesis caused by the downregulation of *Lrp6*, *Tcf7l2*, and *Alpl* might be predominant. On the other hand, the downregulation of *Map2k6* and *Nfatc4* could inhibit osteoclastogenesis. Our qPCR validating analyses of six gene expressions (Figures 5(b)–5(g)) convinced us that RDE could decrease the high bone formation and the bone resorption synchronously in the rat femur, which resulted from ovariectomy and was associated with the canonical Wnt/*β*-catenin signaling, RANKL/RANK signaling. Furthermore, because rats in the RDE group had higher bone mass than those in the OVX group, we inferred that RDE had a more potent inhibitory effect on bone resorption, rather than bone formation.

## 5. Conclusion

RDE can inhibit OVX-induced osteopenia in rats. The mechanism for an antiosteopenic effect of RDE might lie in the synchronous inhibitory effects on both the bone formation and the bone resorption, which is associated with modulating the Wnt/*β*-catenin signaling and the RANKL/RANK signaling. Our study provides evidence that aqueous extract of *Rhizoma Dioscoreae* will have the potential to be used for the treatment of postmenopausal osteoporosis.

## Supplementary Material

Supplementary Material: After treatment of 12 weeks, a total of 168 genes had altered the expression levels (≥1.5-fold) between the distal right femurs from RDE and OVX group rats; that is, 68 genes were upregulated and 100 genes were downregulated. Differentially expressed genes are listed in table below. Columns show the Genbank accession numbers, the genes symbol, P-value and fold change. A positive value of fold change indicates up-regulation of a gene, and a negative value indicates down-regulation. Genes are listed in descending order from absolute value of fold change, respectively.

## Figures and Tables

**Table 1:** Primers used for qPCR analysis.

Genbank ID	Gene name	Forward primer	Reverse primer
NM_002046	*Gapdh *	GGGAAACTGTGGCGTGAT	GAGTGGGTGTCGCTGTTGA
NM_030584	*Sost *	AGTGCCCTTCCTCCTTCTGG	CTGTACTCGGACACGTCTTTGGT
NM_001107892	*Lrp6 *	GCAGGCAGGGTGGAATGA	TTCCGAAGGCTGTGGATAGG
NM_001191052	*Tcf7l2 *	AGCCTACCCATCTTCACTTTCAG	GCTCTCCTTTAGCGTACACTCG
NM_013059	*Alpl *	GCCTGGACCTCATCAGCATT	CGAGAGGGAAGGGTCAGTCAG
NM_053703	*Map2k6 *	GGTAGAAGAGCCGTCTCCACAA	CGCCCTGTAAACCCACCAA
NM_001107264	*Nfatc4 *	GAGAGCGTCCCTCAGAAAACC	TGCTCGTACTGGCTGGGTAAA

**Table 2:** Effect of RDE on BMD and trabecular bone histomorphometric indices after 12 weeks of treatment.

	SHAM	OVX	E2	RDE
BMD (before treatment) (g/cm^2^)	0.210 ± 0.064	0.209 ± 0.068	0.207 ± 0.076	0.208 ± 0.061
BMD (after treatment) (g/cm^2^)	0.305 ± 0.006	0.181 ± 0.006^b^	0.301 ± 0.006^d^	0.280 ± 0.009^c^
BV/TV (%)	33.46 ± 4.89	12.11 ± 3.77^b^	26.45 ± 4.36^d^	18.15 ± 3.34^d^
ES/BS (%)	3.46 ± 0.88	9.73 ± 1.48^b^	4.08 ± 1.63^d^	6.15 ± 1.63^d^
MS/BS (%)	7.90 ± 1.98	13.54 ± 4.99^b^	7.27 ± 2.41^d^	10.82 ± 2.53^d^
MAR, *μ*m/d	1.27 ± 0.36	1.87 ± 0.21^b^	1.24 ± 0.22^d^	1.59 ± 0.25^d^
O.Th, *μ*m	6.16 ± 1.37	7.85 ± 1.79^a^	6.44 ± 1.84^c^	7.59 ± 1.86

Values are presented as the means ± SD (*n* = 12 in each group).

^a^ *P* < 0.05 versus SHAM group; ^b^ *P* < 0.01 versus SHAM group; ^c^ *P* < 0.05 versus OVX group; ^d^ *P* < 0.01 versus OVX group.

**Table 3:** Effect of RDE on PINP and CTX in serum after 12 weeks of treatment.

	SHAM	OVX	E2	RDE
P1NP (*μ*g/L)	7.62 ± 0.13	10.90 ± 0.21^b^	7.38 ± 0.16^d^	8.05 ± 0.27^c^
CTX (*μ*g/L)	12.50 ± 2.31	35.77 ± 4.69^b^	18.77 ± 2.50^d^	25.40 ± 3.64^d^

Values are presented as the means ± SD (*n* = 12 in each group).

^b^ *P* < 0.01 versus SHAM group; ^c^ *P* < 0.05 versus OVX group; ^d^ *P* < 0.01 versus OVX group.

**Table 4:** Effect of RDE on trabecular bone microarchitecture after 12 weeks of treatment.

	SHAM	OVX	E2	RDE
BV/TV (%)	23.60 ± 2.03	2.51 ± 0.48^b^	14.00 ± 0.83^d^	5.09 ± 0.68^c^
Tb.Th (*μ*m)	73.44 ± 0.98	51.61 ± 1.87^b^	71.32 ± 0.92^d^	61.91 ± 0.93^d^
Tb.Sp (*μ*m)	181.62 ± 26.48	678.27 ± 33.21^b^	319.63 ± 95.91^d^	519.29 ± 13.55^c^
Tb.N (1/mm)	3.31 ± 0.33	0.48 ± 0.09^b^	2.05 ± 0.30^d^	0.76 ± 0.05^c^
SMI	1.55 ± 0.14	2.10 ± 0.08^a^	1.72 ± 0.02	1.89 ± 0.02
DA	1.87 ± 0.18	3.81 ± 0.49^b^	2.04 ± 0.05^d^	2.64 ± 0.13^d^

Values are presented as the means ± SD (*n* = 12 in each group).

^a^ *P* < 0.05 versus SHAM group; ^b^ *P* < 0.01 versus SHAM group; ^c^ *P* < 0.05 versus OVX group; ^d^ *P* < 0.01 versus OVX group.

**Table 5:** Top canonical pathways associated with differentially expressed genes between the RDE-H group and OVX group.

Name	*P* value	Number of molecules
Role of osteoblasts, osteoclasts, and chondrocytes in rheumatoid arthritis	6.96*E* − 03	6
Factors promoting cardiogenesis in vertebrates	2.59*E* − 02	3
Role of JAK2 in hormone-like cytokine signaling	2.67*E* − 02	2
Type II diabetes mellitus signaling	5.3*E* − 02	3
Role of macrophages, fibroblasts, and endothelial cells in rheumatoid arthritis	6.16*E* − 02	5

Top canonical pathways for differentially expressed genes were presented. *P* values were calculated by comparing the number of molecules of interest relative to the total number of occurrences of these molecules in all canonical pathways stored in the Ingenuity Pathways knowledge base (Fisher's exact test with *P* value adjusted using the Benjamin-Hochberg multiple testing correction).

## Abbreviations

RD: *Rhizoma Dioscoreae*
RDE: *Rhizoma Dioscoreae extract*
OVX: Ovariectomy
E2: 17*β*-Estradiol
BMD: Bone mineral density
IPA: Ingenuity Pathway Analysis
HPLC: High-performance liquid chromatography
RANKL: Receptor activator of nuclear factor-*κ*B ligand
RANK: Receptor activator of nuclear factor-*κ*B
micro-CT: Microcomputerized tomography
PINP: Procollagen type 1 N-terminal propeptide
CTX: C-Terminal cross-linked telopeptides of type I collagen
HPLC: High-performance liquid chromatography
PTH: Parathyroid hormone
SERMs: Selective estrogen receptor modulator
BV/TV: Bone volume
ES/BS: Eroded surface
MS/BS: Active forming surface
MAR: Mineral apposition rate
O.Th: Osteoid seam width
VOI: Volume of interest
Tb.Th: Trabecular thickness
Tb.Sp: Trabecular separation
Tb.N: Trabecular number
SMI: Structural model index
DA: Degree of anisotropy
SOST: Sclerostin
p38 MAPK: p38 Mitogen-activated protein kinase
LRP5: Low density lipoprotein receptor-related protein 5
LRP6: Low density lipoprotein receptor-related protein 6
TCF/LEF: T cell factor/lymphoid enhancer-binding factor
ALP: Alkaline phosphatase
TNF: Tumor necrosis factor
TRAF6: Tumor necrosis factor receptor-associated factor 6
NFATC1: Nuclear factor of activated T cells C1
NFATC4: Nuclear factor of activated T cells C4.
