# Supplementary material for: Treatment with Rhizoma Dioscoreae Extract Has Protective Effect on Osteopenia in Ovariectomized Rats
Source: ScientificWorldJournal. 2014 Jan 9;2014:645975. doi: 10.1155/2014/645975 (PMC3913014; doi:10.1155/2014/645975)
Supplement: Supplementary file 1 — Supplementary Material: After treatment of 12 weeks, a total of 168 genes had altered the expression levels (≥1.5-fold) between the distal right femurs from RDE and OVX group rats; that is, 68 genes were upregulated and 100 genes were downregulated. Differentially expressed genes are listed in table below. Columns show the Genbank accession numbers, the genes symbol, P-value and fold change. A positive value of fold change indicates up-regulation of a gene, and a negative value indicates down-regulation. Genes are listed in descending order from absolute value of fold change, respectively. [file 645975.f1.doc]

**Supplementary materials**

**Differentially expressed genes between the RDE-H group and OVX group**

| **Genebank Accession** | **Gene Symbol** | ***P*-value** | **Fold Change** |
| --- | --- | --- | --- |
| XM_001057072 | *Skil* | 0.0337 | 2.68 |
| XM_001073983 | *LOC300024* | 0.0340 | 2.52 |
| NM_001130541 | *Colec10* | 0.0161 | 2.45 |
| NM_001109512 | *LOC688765* | 0.0440 | 2.18 |
| NM_001106478 | *Hltf* | 0.0305 | 2.16 |
| NM_001107852 | *Plxna4a* | 0.0426 | 2.10 |
| NM_001039032 | *Lemd2* | 0.0134 | 2.08 |
| XM_001058964 | *Fam184b* | 0.0103 | 2.01 |
| NM_001000348 | *Olr668* | 0.0449 | 1.99 |
| NM_001105848 | *Helz* | 0.0083 | 1.97 |
| XM_001073550 | *LOC686326* | 0.0009 | 1.97 |
| NM_031044 | *Hnmt* | 0.0005 | 1.87 |
| NM_001106856 | *Uba7* | 0.0361 | 1.85 |
| NM_001012164 | *Cd97* | 0.0267 | 1.83 |
| NM_001128493 | *Magebl1* | 0.0441 | 1.82 |
| NM_057107 | *Acsl3* | 0.0221 | 1.81 |
| NM_001107518 | *Nipa2* | 0.0048 | 1.80 |
| XM_001064473 | *LOC683102* | 0.0015 | 1.79 |
| NM_001014121 | *Amz2* | 0.0428 | 1.78 |
| NM_020086 | *Plvap* | 0.0110 | 1.77 |
| XM_342858 | *Adamtsl1* | 0.0261 | 1.77 |
| NM_001107676 | *Proser1* | 0.0368 | 1.77 |
| NM_001000310 | *Olr495* | 0.0369 | 1.75 |
| NM_001015008 | *Tcea3* | 0.0477 | 1.74 |
| XM_001054071 | *LOC679694* | 0.0249 | 1.73 |
| NM_001127494 | *Stk24* | 0.0158 | 1.73 |
| NM_031822 | *Ncoa2* | 0.0488 | 1.71 |
| NM_001172089 | *Gemin5* | 0.0292 | 1.70 |
| XM_001060932 | *LOC682306* | 0.0284 | 1.69 |
| NM_057144 | *Csrp3* | 0.0482 | 1.67 |
| NM_032074 | *Irs3* | 0.0253 | 1.66 |
| NM_001044303 | *Aknad1* | 0.0078 | 1.65 |
| NM_001035007 | *Mbtps2* | 0.0173 | 1.65 |
| NM_145091 | *Pdp2* | 0.0351 | 1.64 |
| NM_001108298 | *Cdc6* | 0.0193 | 1.64 |
| NM_012638 | *Pygm* | 0.0454 | 1.64 |
| NM_001134702 | *Meis1* | 0.0211 | 1.64 |
| XM_001054036 | *LOC679688* | 0.0238 | 1.63 |
| NM_001108594 | *Dtd1* | 0.0127 | 1.63 |
| NM_021769 | *Sult1d1* | 0.0028 | 1.62 |
| XM_001078412 | *Zfp688* | 0.0386 | 1.60 |
| XM_001067433 | *RGD1566396* | 0.0330 | 1.60 |
| XM_001079107 | *RGD1565010* | 0.0462 | 1.60 |
| NM_001025038 | *LOC499465* | 0.0081 | 1.60 |
| NM_001008280 | *Lrrc59* | 0.0141 | 1.60 |
| XM_001059215 | *Eppk1-ps1* | 0.0346 | 1.59 |
| NM_001109134 | *RGD1559896* | 0.0083 | 1.59 |
| NM_001014098 | *Ccdc51* | 0.0267 | 1.59 |
| NM_017350 | *Plaur* | 0.0411 | 1.58 |
| XM_001058172 | *LOC680650* | 0.0495 | 1.58 |
| XM_001069011 | *RGD1566359* | 0.0362 | 1.58 |
| NM_001108745 | *Ppfia2* | 0.0173 | 1.56 |
| NM_001007712 | *Sdpr* | 0.0318 | 1.56 |
| XM_001061142 | *LOC685482* | 0.0394 | 1.56 |
| NM_001109338 | *Slc35f1* | 0.0233 | 1.55 |
| NM_001108585 | *RGD1563222* | 0.0283 | 1.55 |
| NM_022252 | *Slc33a1* | 0.0228 | 1.55 |
| NM_138918 | *Ss18l1* | 0.0135 | 1.55 |
| NM_001008283 | *Mettl23* | 0.0324 | 1.54 |
| XM_001079095 | *LOC691632* | 0.0387 | 1.54 |
| NM_001037655 | *Henmt1* | 0.0474 | 1.54 |
| XM_001060311 | *LOC682184* | 0.0132 | 1.53 |
| NM_001015016 | *Cd72* | 0.0480 | 1.53 |
| XR_086029 | *LOC691889* | 0.0004 | 1.53 |
| NM_147142 | *LOC257650* | 0.0314 | 1.51 |
| XM_001071717 | *RGD1562066* | 0.0247 | 1.51 |
| NM_001108750 | *Cpne8* | 0.0152 | 1.50 |
| NM_001130562 | *Samd12* | 0.0112 | 1.50 |
| NM_001108742 | *Slc41a2* | 0.0150 | -2.38 |
| NM_031239 | *Sh3gl1* | 0.0108 | -2.33 |
| XM_001067479 | *LOC683790* | 0.0424 | -2.29 |
| NM_022175 | *Rhox5* | 0.0235 | -2.23 |
| NM_001008814 | *Kb21* | 0.0479 | -2.21 |
| NM_001013878 | *Fam149b1* | 0.0407 | -2.21 |
| NM_001107548 | *Usp31* | 0.0125 | -2.19 |
| NM_001107042 | *Hoxb3* | 0.0198 | -2.04 |
| NM_019367 | *Ppt2* | 0.0174 | -2.03 |
| NM_001191052 | *Tcf7l2* | 0.0249 | -2.03 |
| NM_031753 | *Alcam* | 0.0245 | -2.02 |
| NM_001106761 | *Brf1* | 0.0483 | -2.02 |
| XM_001068568 | *LOC689833* | 0.0163 | -2.01 |
| NM_212491 | *Wdr46* | 0.0027 | -1.98 |
| XM_001060182 | *LOC682160* | 0.0345 | -1.97 |
| NM_001107569 | *Bbs1* | 0.0066 | -1.96 |
| NM_012766 | *Ccnd3* | 0.0284 | -1.92 |
| NM_030584 | *Sost* | 0.0440 | -1.91 |
| NM_001109362 | *Iqcf6* | 0.0227 | -1.91 |
| NM_173319 | *Vom2r31* | 0.0224 | -1.90 |
| NM_012624 | *Pklr* | 0.0047 | -1.89 |
| NM_001009602 | *Actr10* | 0.0246 | -1.87 |
| NM_021764 | *Rbck1* | 0.0083 | -1.86 |
| NM_001108052 | *Rin3* | 0.0289 | -1.86 |
| NM_001107267 | *Lats2* | 0.0031 | -1.86 |
| NM_001109515 | *Slc25a28* | 0.0472 | -1.85 |
| XM_001076316 | *RGD1563757* | 0.0317 | -1.85 |
| NM_001145273 | *LOC688869* | 0.0078 | -1.85 |
| XM_001055076 | *LOC679177* | 0.0339 | -1.83 |
| XM_001065372 | *LOC291249* | 0.0165 | -1.83 |
| NM_153316 | *Slc35e4* | 0.0026 | -1.81 |
| XM_001069414 | *LOC684208* | 0.0391 | -1.79 |
| XM_001071133 | *Ccnf-ps1* | 0.0256 | -1.79 |
| NM_001107475 | *Suv420h2* | 0.0120 | -1.78 |
| XM_573687 | *LOC498435* | 0.0063 | -1.78 |
| NM_080480 | *Pip4k2c* | 0.0481 | -1.75 |
| XM_001059906 | *Smg5* | 0.0462 | -1.74 |
| NM_001127654 | *Tm6sf2* | 0.0127 | -1.74 |
| NM_001173509 | *Art4* | 0.0428 | -1.73 |
| NM_001014099 | *Paqr8* | 0.0188 | -1.73 |
| NM_001109456 | *Sec24c* | 0.0484 | -1.73 |
| NM_001107333 | *Papd7* | 0.0181 | -1.72 |
| NM_001002835 | *Smoc1* | 0.0387 | -1.72 |
| NM_138613 | *Rnf112* | 0.0376 | -1.72 |
| NM_001008370 | *Rab1b-ps1* | 0.0277 | -1.72 |
| NM_001106295 | *Tufm* | 0.0304 | -1.70 |
| XM_229242 | *LOC316820* | 0.0408 | -1.70 |
| NM_001000412 | *Olr867* | 0.0499 | -1.70 |
| NM_001108355 | *Cnot6l* | 0.0466 | -1.70 |
| NM_001005871 | *Atp2b4* | 0.0181 | -1.70 |
| NM_053337 | *Pias2* | 0.0030 | -1.69 |
| NM_001025278 | *Pus10* | 0.0275 | -1.69 |
| NM_001107415 | *Sall1* | 0.0441 | -1.67 |
| XM_001055993 | *LOC502167* | 0.0283 | -1.67 |
| NM_012828 | *Cacnb3* | 0.0026 | -1.67 |
| NM_001014025 | *RGD1305014* | 0.0166 | -1.66 |
| NM_001109607 | *Hint1* | 0.0203 | -1.66 |
| XM_001075175 | *RGD1565048* | 0.0146 | -1.65 |
| NM_001017478 | *Cxcl16* | 0.0228 | -1.65 |
| NM_181823 | *Rhot2* | 0.0179 | -1.65 |
| NM_001108155 | *Kif23* | 0.0243 | -1.64 |
| NM_001001718 | *Rai12* | 0.0338 | -1.64 |
| NM_001047880 | *Slc25a15* | 0.0091 | -1.63 |
| NM_022206 | *Unc5a* | 0.0430 | -1.63 |
| NM_001191989 | *Dtx3* | 0.0339 | -1.63 |
| NM_021751 | *Prom1* | 0.0397 | -1.61 |
| NM_001013854 | *Bod1* | 0.0277 | -1.60 |
| NM_001106845 | *Ankrd34c* | 0.0464 | -1.59 |
| NM_001037219 | *Foxk1* | 0.0114 | -1.59 |
| NM_001107892 | *Lrp6* | 0.0394 | -1.59 |
| NM_001107264 | *Nfatc4* | 0.0402 | -1.58 |
| XM_001061779 | *Cd300lg* | 0.0074 | -1.58 |
| NM_001108779 | *Tmem115* | 0.0272 | -1.58 |
| XM_001055615 | *A26c2* | 0.0429 | -1.58 |
| XM_345686 | *RGD1559639* | 0.0330 | -1.58 |
| NM_001134570 | *RGD1560258* | 0.0004 | -1.58 |
| XM_001065777 | *LOC685924* | 0.0172 | -1.57 |
| NM_133418 | *Slc25a10* | 0.0281 | -1.57 |
| XM_226511 | *RGD1564626* | 0.0474 | -1.57 |
| NM_001105751 | *Hivep1* | 0.0396 | -1.57 |
| NM_181362 | *Cand2* | 0.0445 | -1.56 |
| XM_001060709 | *LOC682258* | 0.0085 | -1.56 |
| XM_001072370 | *LOC689881* | 0.0411 | -1.56 |
| NM_031521 | *Ncam1* | 0.0352 | -1.56 |
| NM_139088 | *Impg2* | 0.0399 | -1.55 |
| NM_001008803 | *Krt82* | 0.0391 | -1.54 |
| NM_001005246 | *Dmd* | 0.0266 | -1.54 |
| NM_001110838 | *Toag1* | 0.0140 | -1.54 |
| XM_001054040 | *LOC679690* | 0.0191 | -1.54 |
| NM_001106907 | *Tgfbrap1* | 0.0127 | -1.54 |
| NM_001017537 | *Tex261* | 0.0199 | -1.53 |
| NM_053643 | *Cds2* | 0.0296 | -1.53 |
| NM_001106440 | *Apoa1bp* | 0.0325 | -1.52 |
| NM_153735 | *Nptx1* | 0.0121 | -1.52 |
| NM_001177368 | *Lrrn2* | 0.0463 | -1.52 |
| NM_001106300 | *Ate1* | 0.0168 | -1.51 |
| XM_001078888 | *LOC687513* | 0.0230 | -1.51 |
| NM_013059 | *Alpl* | 0.0093 | -1.50 |
| NM_053703 | *Map2k6* | 0.0156 | -1.50 |
| XM_001066042 | *LOC683463* | 0.0144 | -1.50 |
